# Transcriptional Repressor NIR Functions in the Ribosome RNA Processing of Both 40S and 60S Subunits

**DOI:** 10.1371/journal.pone.0031692

**Published:** 2012-02-20

**Authors:** Jianguo Wu, Ying Zhang, Yingshuang Wang, Ruirui Kong, Lelin Hu, Roland Schuele, Xiaojuan Du, Yang Ke

**Affiliations:** 1 Key Laboratory of Carcinogenesis and Translational Research (Ministry of Education), Genetics Laboratory, Peking University School of Oncology, Beijing Cancer Hospital & Institute, Beijing, China; 2 Department of Cell Biology, School of Basic Medical Sciences, Peking University Health Science Center, Beijing, China; 3 Medical Research Center, Freiburg University, Freiburg, Germany; University of Texas Health Science Center at San Antonio/Greehey CCRI, United States of America

## Abstract

**Background:**

NIR was identified as an inhibitor of histone acetyltransferase and it represses transcriptional activation of p53. NIR is predominantly localized in the nucleolus and known as Noc2p, which is involved in the maturation of the 60S ribosomal subunit. However, how NIR functions in the nucleolus remains undetermined. In the nucleolus, a 47S ribosomal RNA precursor (pre-rRNA) is transcribed and processed to produce 18S, 5.8S and 28S rRNAs. The 18S rRNA is incorporated into the 40S ribosomal subunit, whereas the 28S and 5.8S rRNAs are incorporated into the 60S subunit. U3 small nucleolar RNA (snoRNA) directs 18S rRNA processing and U8 snoRNA mediates processing of 28S and 5.8 S rRNAs. Functional disruption of nucleolus often causes p53 activation to inhibit cell proliferation.

**Methodology/Principal Findings:**

Western blotting showed that NIR is ubiquitously expressed in different human cell lines. Knock-down of NIR by siRNA led to inhibition of the 18S, 28S and 5.8S rRNAs evaluated by pulse-chase experiment. Pre-rRNA particles (pre-rRNPs) were fractionated from the nucleus by sucrose gradient centrifugation and analysis of the pre-RNPs components showed that NIR existed in the pre-RNPs of both the 60S and 40S subunits and co-fractionated with 32S and 12S pre-rRNAs in the 60S pre-rRNP. Protein-RNA binding experiments demonstrated that NIR is associated with the 32S pre-rRNA and U8 snoRNA. In addition, NIR bound U3 snoRNA. It is a novel finding that depletion of NIR did not affect p53 protein level but de-repressed acetylation of p53 and activated p21.

**Conclusions:**

We provide the first evidence for a transcriptional repressor to function in the rRNA biogenesis of both the 40S and 60S subunits. Our findings also suggested that a nucleolar protein may alternatively signal to p53 by affecting the p53 modification rather than affecting p53 protein level.

## Introduction

In the nucleolus of mammalian cells RNA polymerase I transcribes a 47S ribosomal RNA precursor (pre-rRNA) which contains a 5′ external transcribed spacer (5′-ETS), followed by the 18S rRNA, internal transcribed spacer 1 (ITS1), 5.8S rRNA, internal transcribed spacer 2 (ITS2), 28S rRNA and the 3′ external transcribed spacer (3′-ETS). Upon synthesis, the 47S pre-rRNA transcript is modified by ribose methylation and pseudouridine conversion and cleaved at specific sites to generate a series of intermediates and consequently produce matured 18S, 28S, and 5.8S rRNAs. Several cleavage pathways have been described for processing of the pre-rRNA to produce the matured rRNAs and at least two cleavage pathways have been described in mammalian cells ([1] [2]). The 18S rRNA is incorporated into the 40S ribosomal subunit, whereas the 28S and 5.8S rRNAs are incorporated into the 60S ribosomal subunit with the 5S rRNA which is transcribed by RNA polymerase III outside of the nucleolus.

Modifications and cleavages of pre-rRNA are directed by small nucleolar RNAs (snoRNAs) [3], [4]. U3 snoRNA nucleotide base pairs with sequences in the 5′ ETS and ITS-1 blanking 18S rRNA in the 47S rRNA and mediates cleavage at A0, A1 and A2 sites and is required for 18S rRNA processing [5], [6], [7]. U3 snoRNA-associated proteins (UTPs) play essential roles in 40S subunit biogenesis and are main components of small subunit (SSU) processome. The SSU components possess the following characteristics: they are nucleolar, associated with U3 snoRNA and are required for 18S rRNA processing. Upon cleavage at A2 site, SSU together with the 18S rRNA departs from the transcribed rRNA as the 40S pre-RNPs and 60S subunit rRNA processing factors are recruited to the remaining 32S pre-rRNA to form the large subunit processome (LSU) to fulfill the cleavage of 32S pre-rRNA to produce 28S rRNA and 5.8S rRNA [8]. Up to date, U8 snoRNA is identified as the only snoRNA required for 28S and 5.8S rRNA processing [9], [10]. U8 binds 32S rRNA and may function as a chaperone for 32S pre-rRNA folding and facilitate the 28S and 5.8S rRNA processing [11]. The *Xenopus* homologues of the LSm (like Sm) proteins including LSm2, -3, -4, -6, -7, and -8 have been identified as U8 binding proteins and the presence of LSm8 was considered to be consistent with the nuclear localization of U8 [12]. A *Xenopus* 29 kDa protein (X29) binds U8 RNA [13] and is capable of removing the m^227^G cap from U8 RNA, which may lead to degradation of U8 RNA resulting in an inhibition of pre-rRNA processing [14]. A mammalian DEAD box protein Ddx51 promotes the release of U8 snoRNA from pre-rRNA and acts in 3′ end maturation of 28S rRNA [15].

For the 60S ribosome subunit biogenesis, three down-stream genes of onco-protein *myc* including Bop1, Pes1 and WDR12 have been identified to play key roles in the processing of 28S and 5.8S rRNAs in mammalian cells. Bop1 was the first identified mammalian protein being involved in the processing of 28S and 5.8S rRNAs and functioning in cell proliferation [16], [17]. Pes1 was found to physically and functionally interact with Bop1 to form a Bop1-Pes1 complex [2], [18], [19] and WDR12 has been demonstrated to form the PeBoW complex with Bop1-Pes1 to function in the 28S rRNA and 5.8S rRNA processing and cell proliferation [20]. Bop1, Pes1 and WDR12 are interdependent and play key roles in cell proliferation and 60S ribosome subunit biogenesis [21]. However, none of them has been found to bind 32S rRNA or U8 snoRNA.

Ribosome biogenesis governs protein synthesis and cell proliferation, is thus tightly controlled. Disruptions of nucleolar function caused by chemical reagents or deficiency of nucleolar proteins have been shown to generate nucleolar stress signaling to p53. For instance, low concentrations (5–10 nM) of actinomycin D selectively inhibit RNA pol I dependent transcription and stimulates stabilization of p53 [22] [23]. p53 is activated by the chemotherapeutic agent 5-Fluorouracil (5-FU) which blocks pre-rRNA processing by incorporating newly synthesized rRNA. A dominant negative mutant of Bop1 inhibits ribosomal biogenesis and elicits p53 activation [24]. Recently, it was found that disruption of human UTP18 and hUTP14a inhibited 18S rRNA processing and induced p53 activation [25], [26]. All these findings identify p53 as a molecule which is critical in sensing nucleolar stress.

In unstressed cells, the p53 protein level remains low through regulation of its protein stability by a number of negative regulators. MDM2 serves as a key negative feedback regulator for p53 and various stresses activate distinct cellular signaling pathways leading to the suppression of MDM2 activity and activation of p53 [27] [28]. Thus, the p53-MDM2 feedback loop plays an essential role in response to a multitude of genotoxic and cytotoxic stressors. Several ribosomal large subunit proteins including RPL11 [29] [30], RPL23 [31] [32] and RPL5 [33] and a small subunit protein RPS7 [34] [35] have been found to interact with MDM2. This binding inhibits the MDM2 E3 ligase function, resulting in p53 accumulation and activation.

Our previous study identified 1A6/DRIM as the human UTP20 which functions in the pol I transcription and 18S rRNA processing [36], [37]. A tandem affinity purification (TAP) experiment found that 1A6/DRIM exists in the TAP-NIR complex (our unpublished data). NIR has been identified as a novel INHAT (inhibitor of histone acetyltransferase) which represses p53 transcription activation and a negative regulator of TAp63 [38] and represents a novel HDAC-independent inhibitor of histone acetyltransferase [39]. Jain and colleagues found that Aurora B binds to NIR to form an Aurora-NIR-p53 complex and inhibits p53 activation [40]. *In vitro* study found that Aurora B phosphorylates multiple sites in the p53 DNA-binding domain [40]. NIR is also known as the homologue of yeast Noc2p which was reported to be involved in ribosome assembly and intranuclear and nucleocytoplasmic transport of pre-ribosomal particles [41], [42]. However, how NIR functions in the nucleolus remains undetermined.

In the present study, we showed that NIR is required for 18S, 28S and 5.8S rRNA processing and further investigated the mechanisms by which NIR functions in the rRNA processing. We also found an alternative pathway for p53 activation caused by depletion of NIR.

## Results

### NIR is expressed in the nucleolus of various human cell lines

**Figure 1:**
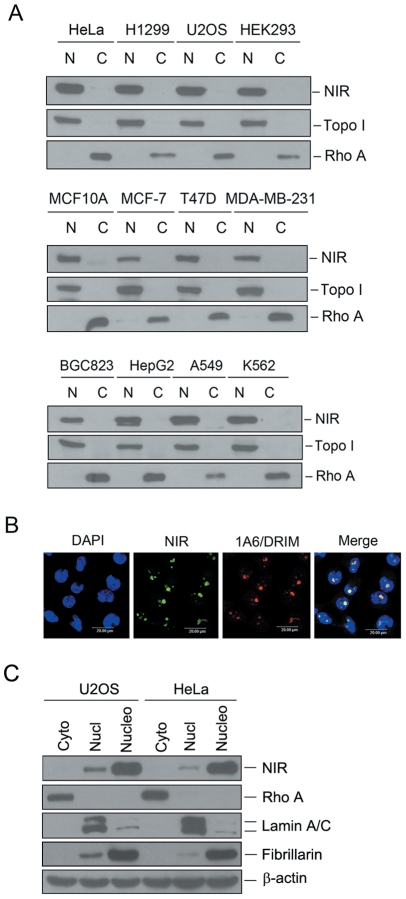
NIR is expressed in different human cell lines and mainly localized to the nucleolus. A. Cytosolic and nuclear extracts were fractionized from multiple cancer cell lines as indicated at the top of blot. Same amount of protein was separated on SDS-PAGE, and transferred onto PVDF membranes. Blots were probed with anti-NIR. Fractionation was controlled by using nuclear marker protein topoisomerase I (Topo I) and a cytosolic marker protein RhoA. N represents nuclear extract and C represents cytoplasmic extract. B. Indirect immunofluorescence was performed with anti-NIR polyclonal antibody. NIR specific signal was recognized with FITC-conjugated goat anti-rabbit IgG. As a nucleolar protein, 1A6/DRIM was detected with anti-1A6/DRIM monoclonal antibody. 1A6/DRIM specific signal was recognized with TRITC-conjugated goat anti-mouse IgG. Nucleus was stained with DAPI. The image was obtained with confocal microscopy. C. Cytoplasmic, nucleoplasmic and nucleolar lysates were fractionized from U2OS and HeLa cells respectively. Same amount of protein from the above lysates was separated on SDS-PAGE, and transferred onto PVDF membranes. Blots were probed with anti-NIR. Rho A, lamin A/C and fibrillarin were used as controls for cytoplasmic, nucleoplasmic and nucleolar fractions respectively. Beta-actin was used as a loading control.

To evaluate the function of NIR protein and to detect endogenous NIR expression, we analyzed the expression of NIR in various human cell lines. Cellular fractions were prepared and proteins from the fractions were subjected to Western blotting. Figure 1A shows that NIR was ubiquitously expressed in the nuclear extracts of the cell lines under evaluation. Subcellular localization of endogenous NIR was also determined by indirect immunofluorescence performed with the polyclonal anti-NIR antibody while an anti-1A6/DRIM monoclonal antibody was used as a nucleolar protein marker. As shown in Figure 1B, NIR was predominantly localized in the nucleolus and co-localized with 1A6/DRIM. To confirm the nucleolar localization of endogenous NIR, we fractionized cytoplasmic, nucleoplasmic and nucleolar lysate from U2OS and HeLa cells and NIR protein was detected by Western blotting with the above fractions. Figure 1C shows that cytoplasmic protein Rho A was only detected in the cytoplasmic lysate and Lamin A/C was mainly found in the nucleoplasm. Fibrillarin was used as a nucleolar protein control. NIR protein shows the same localization as fibrillarin which is mainly localized in the nucleolus and detectable signal was also found in the nucleoplasm.

### NIR is required for processing of 18S, 28S and 5.8S rRNAs

**Figure 2:**
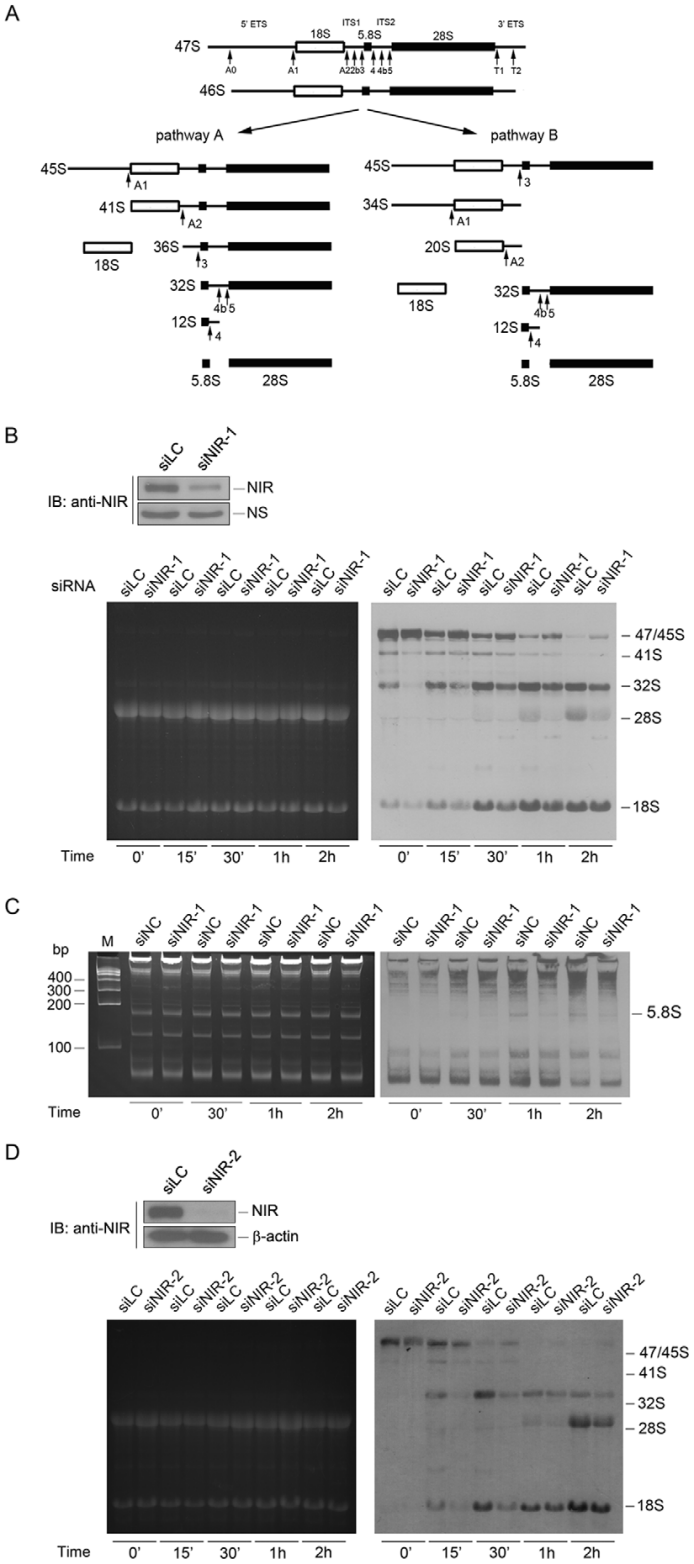
Knockdown of NIR resulted in inhibition of 18S, 28S and 5.8S rRNA processing. A. Schematic representation of rRNA processing pathways in HeLa cells. B. U2OS cells were transfected with a NIR-specific siRNA (siNIR-1) or luciferase siRNA (siLC) as a control. At 72 hrs post-transfection, cell lysates were prepared. Proteins from the lysates were separated on SDS-PAGE, transferred onto a PVDF membrane. Blot was probed for detection of NIR protein (upper panel). NS, non-specific band. Above siRNA transfected U2OS cells were labeled with L-[*methyl*-^3^H] methionine and RNA was extracted at indicated time points. Equal amount of RNA from each sample was resolved on a 1% agarose gel. The gel was stained with ethidium bromide (EB) for photography (lower left panel) and transferred to a membrane for autoradiography (lower right panel). C. U2OS cells were transfected with siRNAs as in B. At 72 hrs post-transfection, cells were labeled with 3 µCi/ml [5, 6-^3^H] uridine for 30 min. RNA was extracted at indicated time points. Equal amount of RNA from each sample was resolved on a 10% polyacrylamide/7.5 M urea gel. The gel was stained with ethidium bromide (EB) for photography (left panel) and transferred to a membrane for autoradiography (right panel). D. U2OS cells were transfected with a second NIR-specific siRNA (siNIR-2) or luciferase siRNA (siLC) as a control. At 72 hrs post-transfection, cell lysates were prepared and subjected to Western blotting for detection of NIR protein (upper panel). Beta-actin was used as a loading control. Above siRNAs (siNIR-2 and siLC) transfected U2OS cells were labeled with L-[*methyl*-^3^H] methionine and RNA was extracted at indicated time points. Equal amount of RNA from each sample was resolved on a 1% agarose gel. The gel was stained with ethidium bromide (EB) for photography (lower left panel) and transferred to a membrane for autoradiography (lower right panel).

To evaluate whether NIR is required for rRNA processing, a NIR-specific siRNA (small interference RNA, siNIR-1) was transfected into U2OS cells to knock down NIR expression and newly synthesized rRNA was analyzed with pulse-chase experiment. Schematic representation of rRNA processing pathways is shown in Figure 2A. Figure 2B shows the pulse-chase result. The 41S rRNA level was decreased and the 47S pre-rRNA was accumulated in NIR-depleted cells indicating that rRNA processing was inhibited at A1. Accordingly, the 18S rRNA level was decreased at 0 h, 15 min, 30 min and 1 h of chase. These results suggested that pathway A was inhibited by NIR depletion. However, we cannot judge if pathway B was affected under our experiment condition. It is notable that the 32S rRNA level dramatically decreased at 0 min, and 15 min of chase and was still inhibited at 30 min, 1 h and 2 h of chase time. The mature 28S rRNA is produced at 1 h and 2 h of chase in control siRNA treated cells, whereas it was lacked at 1 h of chase and only a faint band for 28S rRNA was detected at 2 h of chase in the NIR depleted cells. It indicated that the processing from 41S rRNA to 32S rRNA was inhibited, while the cleavage from 32S to 28S rRNA was blocked. Since both of the mature 28S and 5.8S rRNA are cleaved from 32S rRNA precursor, we next examined the newly synthesized 5.8S rRNA level. NIR-specific siRNA-1 was transfected into U2OS cells, cells were labeled with [5, 6-^3^H] uridine and total RNA extracted at different time periods was resolved on a 10% polyacrylamide/7.5 M urea gel. As shown in Figure 2C, newly synthesized 5.8S rRNA decreased at 1 h and 2 h of chase in NIR depleted cells. To minimize the potential off-target effect of NIR siRNA, a second siRNA targeting NIR (siNIR-2) was transfected into U2OS cells and pulse chase experiment was performed to evaluate the effect of NIR knockdown on 18S and 28S rRNA processing. As shown in Figure 2D, 18S rRNA and 28S rRNA processing was inhibited when NIR was silenced with siNIR-2. Collectively, these results indicated that silencing of NIR expression resulted in the inhibition of 18S, 28S and 5.8S rRNA processing.

### NIR is present in both of the pre-60S and pre-40S ribosomal particles and co-sediment with the 32S and 12S pre-rRNAs

**Figure 3:**
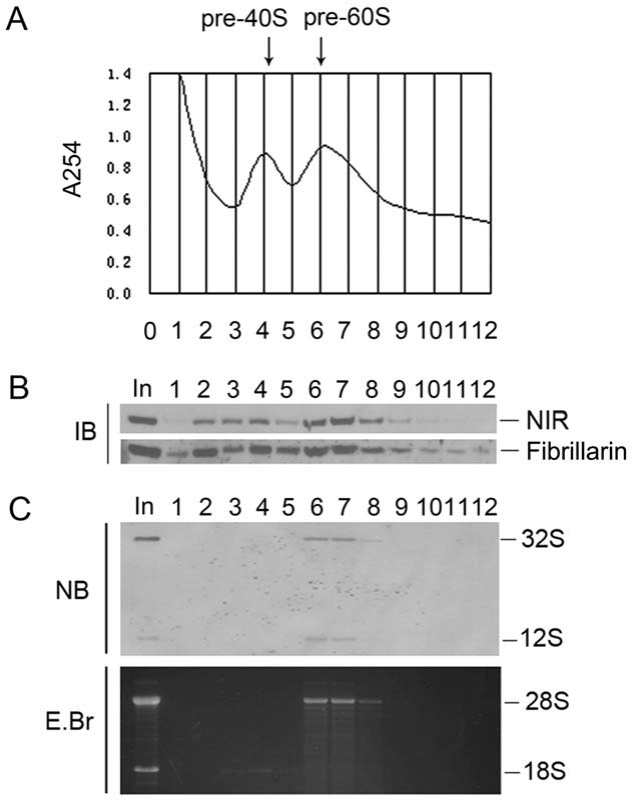
NIR is present in both of the 40S pre-rRNP and 60S pre-rRNP and co-sediment with 32S and 12S rRNA precursors in the nucleolus. A. Nuclear extracts were prepared from U2OS cells and fractionized on 10% to 40% sucrose density gradient. The absorbance at 254 nm (A_254_) of each fraction was profiled and the position of pre-ribosomal subunits was indicated. B. Proteins from fractions described in A were separated on a SDS-PAGE and subjected to immunoblotting analysis using anti-NIR antibody. Fibrillarin was probed as a control. C. RNA from each fraction was resolved on a 1% agarose-glyoxal gel and transferred onto nylon membrane after stained with EB (lower panel). Blot was probed with biotin-labeled ITS-2 oligonucleotide (upper panel). In, un-fractionized nuclear extract.

Eukaryotic rRNA processing occurs coordinately with the assembly of pre-ribosomal particles (pre-rRNP) in the nucleolus. To further confirm the participation of NIR in the rRNA processing, pre-rRNP particles were fractionized by applying nuclear extracts on sucrose gradient centrifugation. As shown in Figure 3A, fraction 3 to 5 represents the pre-40S particle and fraction 6 to 8 represents the pre-60S particle. Protein and RNA from each fraction were analyzed in parallel. One half of each fraction was analyzed for the presence of NIR protein with Western blotting (Figure 3B). Fibrillarin is known to be present in both of the pre-40S RNP and pre-60S RNP and was used as control. Total RNA was extracted from the remaining half of each fraction and resolved on 1% agarose-glyoxal gel to determine the presence of 18S and 28S rRNAs in the pre-rRNP (Figure 3C, lower panel). It showed that 18S rRNA mainly sediment in pre-40S particles in fraction 3 to 5 and 28S rRNA sediment in pre-60S particle in fraction 6 to 8. Figure 3B showed that NIR was predominantly present in fraction 2 to 4 and fractions 6 to 8 in the nuclear extracts, demonstrating NIR existed in both of pre-40S and pre-60S particles. These data further confirmed that NIR participated in 60S and 40S subunit biogenesis. To evaluate presence of 32S and 12S rRNA in the pre-RNPs, the RNA in Figure 3C (the lower panel) was blotted from the agarose gel onto a nylon membrane and blot was probed with a biotin-labeled DNA fragment from the ITS2 region. The results showed that 32S and 12S rRNA precursors existed in fraction 6 to 8 (Figure 3C, upper panel). Taken together, it demonstrated that NIR co-sediment with the 32S and 12S rRNAs in the nucleolus suggesting that NIR may be associated with 32S and 12S pre-rRNAs.

### NIR is associated with 32S and 12S pre-rRNAs and U8 snoRNA *in vivo*

**Figure 4:**
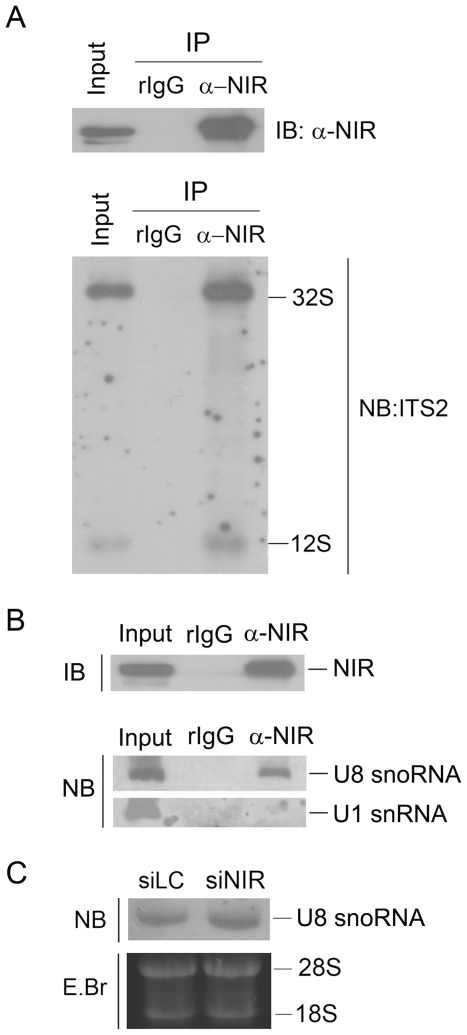
NIR is associated with 32S and 12S pre-rRNAs and U8 snoRNA *in vivo* and knock down of NIR did not affect U8 snoRNA. A. Immunoprecipitation was performed with anti-NIR antibody on U2OS cell lysates, protein and RNA were prepared in parallel. Proteins were separated on SDS-PAGE, blotted onto a PVDF membrane and subjected to Western blotting analysis probed with anti-NIR (upper panel). Ten percent of cell lysates were loaded as input control. RNA extracted from the immunoprecipitation was resolved on 1% agarose-glyoxal gel and blotted onto a nylon membrane. Blot was probed with biotin-labeled ITS-2 oligonucleotide (lower panel). B. Immunoprecipitation was performed with anti-NIR antibody and proteins from the immunoprecipitation were subjected to Western blotting analysis probed with anti-NIR as described in A (upper panel). Ten percent of cell lysates were loaded as input control. RNA extracted from the immunoprecipitation was resolved on 7% polyacrylamide/8.3 M urea and blotted onto a nylon membrane. Blot was probed with biotin-labeled U3 snoRNA-specific RNA probe or U1-specific RNA probe (lower panel). RNA extracted from 2.5% cell lysates were loaded as input control. C. U2OS cells were transfected with NIR specific siRNA (siNIR) or a siRNA targeting luciferase (siLC) as a control. Seventy-two hours post transfection, total RNA was extracted. Equal amount RNA was resolved on a 7% polyacrylamide–8.3 M urea gel and transferred onto a nylon membrane. Blot was hybridized with U8 snoRNA specific probe.

To investigate if NIR is associated with the 32S rRNA and 12S rRNA, cell lysates was prepared from U2OS cells and immunoprecipitation was performed with anti-NIR antibody. Proteins from one half of the immunoprecipitation were subjected to Western blotting for evaluation of NIR. RNA extracted from the remaining half of the immunoprecipitation was analyzed by Northern blotting probed with an ITS-2 probe. The results showed that both of 32S and 12S rRNAs existed in the NIR-specific immunoprecipitates (Figure 4A). This analysis demonstrated that NIR was associated with both 32S rRNA and 12S rRNA *in vivo*.

Our above findings prompted us to investigate if NIR interacts with U8 snoRNA, which is known to bind 32S rRNA and facilitate 28S and 5.8S rRNA processing. To address this issue, immunoprecipitation was performed with anti-NIR antibody on the cell lysates extracted from U2OS cells. Proteins from one half of the immunoprecipitation were subjected to Western blotting for evaluation of NIR. RNA extracted from the remaining half of the immunoprecipitate was subjected to Northern blotting hybridized with a biotin-labeled U8 snoRNA-specific probe. As shown in Figure 4B (lower panel), U8 snoRNA was present in the NIR-specific immunocomplex while U1 which is required for mRNA splicing did not exist in the NIR-specific immunocomplex demonstrating that NIR was specifically associated with U8 snoRNA. Given that U8 snoRNA is the only known snoRNA required for 28S and 5.8S rRNA processing, we next wanted to know if knock down of NIR affected U8 snoRNA level. To this end, a NIR specific siRNA was transfected into U2OS cell, RNA was extracted 72 hours after transfection and subjected to Northern blotting for evaluation of U8 snoRNA. As shown in Figure 4C, U8 snoRNA was not changed by knockdown of NIR. These results demonstrated that depletion of NIR inhibited 28S and 5.8S rRNA processing without affecting U8 snoRNA level.

### NIR is associated with U3 snoRNA

**Figure 5:**
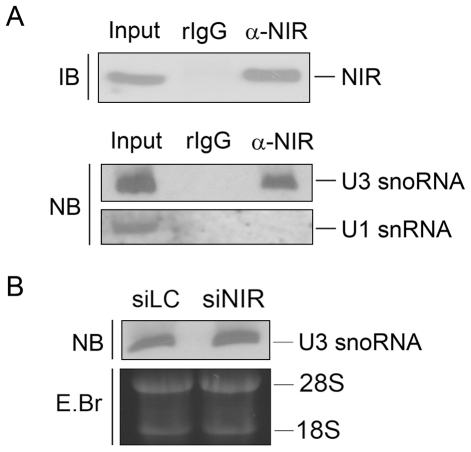
NIR was associated with U3 snoRNA *in vivo* and knock down of NIR didn't affect U3 snoRNA level. A. Immunoprecipitation was performed with anti-NIR antibody and whole cell lysates from U2OS cells. Proteins from NIR immunoprecipitates were separated on a SDS-PAGE and blotted onto a PVDF membrane. Blot was probed with anti-NIR antibody. Ten percent of the lysates were loaded as input control (upper panel). RNA was extracted from the above immunoprecipitation, resolved on a 7% polyacrylamide–8.3 M urea gel and blotted onto a nylon membrane. The blot was probed with biotin-labeled U3 snoRNA-specific probe or U1-specific RNA probe (lower panel). B. U2OS cells were transfected with NIR specific siRNA (siNIR) or a siRNA targeting luciferase (siLC) as a control. Seventy-two hours post transfection, total RNA was extracted. Equal amount RNA was resolved on a 7% polyacrylamide–8.3 M urea gel and transferred onto a nylon membrane. Blot was hybridized with U3 snoRNA specific probe.

U3 snoRNA base pairs with 47S rRNA and facilitates processing of 18S rRNA. Figure 2 showed that both of 41S rRNA and 18S rRNA levels were inhibited by NIR depletion, suggesting the function of NIR in 18S rRNA processing may be related to U3 snoRNA. We therefore examined association between human NIR and U3 snoRNA. Immunoprecipitation was performed on U2OS cell lysates with anti-NIR antibody. RNA was extracted from the immunoprecipitation and the co-precipitated U3 snoRNA was analyzed with Northern blotting. As shown in Figure 5A, U3 snoRNA was present in the NIR specific immunoprecipitates while U1, as a control was not present in the NIR-immunoprecipitates. The results indicated that NIR was associated with U3 snoRNA *in vivo*. What we next wanted to know was if depletion of NIR affected U3 snoRNA level. NIR specific siRNA was transfected into U2OS cell and total RNA was extracted and subjected to Northern Blotting hybridized with a U3 snoRNA probe. The same amount of RNA was resolved on a 1% agarose gel and stained with EB (Figure 5B, lower panel) as a loading control. As shown in Figure 5B, U3 snoRNA level was not altered by knockdown of NIR. The results demonstrated that inhibition of 18S rRNA processing was caused by NIR expression deficiency.

### Depletion of NIR de-repressed acetylation of p53 and activated p53

**Figure 6:**
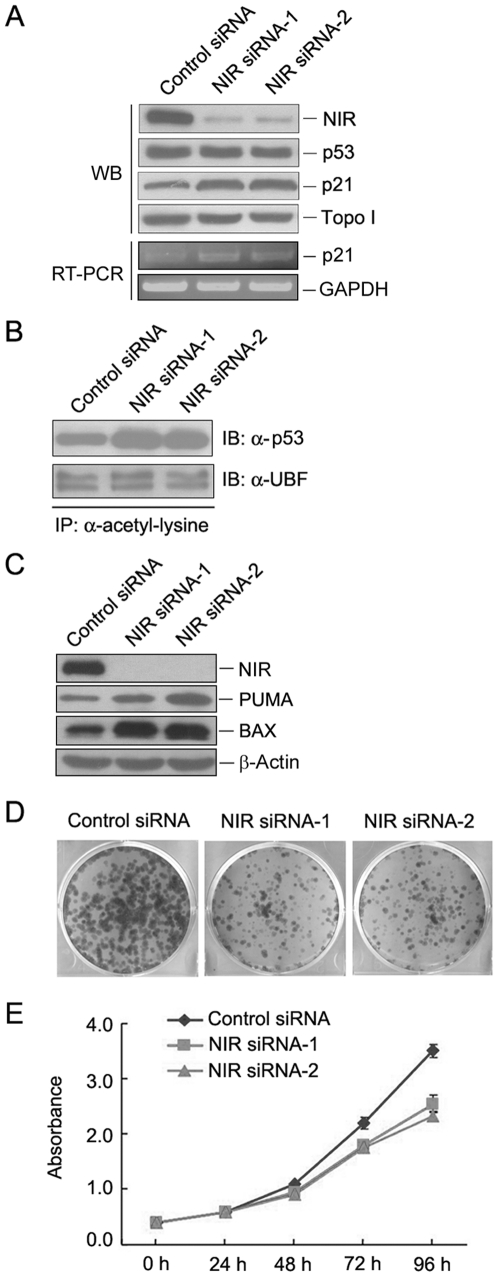
Depletion of NIR may activate p53 by de-repressing p53 acetylation. A. U2OS cells were transfected with NIR-specific siRNA or control siRNA respectively. At 72 hrs post transfection, cell lysates were prepared. Proteins from the lysates were separated on SDS-PAGE, transferred onto PVDF membrane. Blot was probed for detection of NIR, p53 and p21. Topoisomerase I (Topo I) was used as a loading control. RT-PCR was performed with RNA extracted from the above siRNA transfected cells for evaluation of p21 mRNA. Amplification of GAPDH was used as a loading control. B. U2OS cells were transfected with NIR-specific siRNA or control siRNA respectively. At 72 hrs post transfection, cell lysates were prepared. Immunoprecipitation was performed with anti-acetyl-lysine antibody on the cell lysates. Proteins from the immunoprecipitates were separated on SDS-PAGE and transferred onto a PVDF membrane. Blot was probed with anti-p53 or anti-UBF antibody. C. U2OS cells were transfected with NIR-specific siRNA or control siRNA respectively. At 72 hrs post transfection, cell lysates were prepared. Proteins from the lysates were separated on SDS-PAGE, transferred onto PVDF membrane. Blot was probed for detection of NIR, PUMA and BAX. Beta-actin was used as a loading control. D. U2OS cells were transfected with NIR siRNA or control siRNA respectively. Two thousand cells were seeded in 6 mm plates 72 hrs post-transfection. Cells were grown for 10 days and cell colonies were stained with Coomassie Bright Blue after fixation with ethanol/acetic acid. E. U2OS cells were transfected with NIR siRNA or control siRNA respectively. Cells were seeded in 96-well plates 72 hrs post transfection. Ten microliters of CCK-8 was added to the cells and absorbance at 450 nm was measured at different time periods. The experiment was repeated three times in duplicate and growth curves were plotted using absorbance at 450 nm vs. different time points.

To determine whether depletion of NIR activates p53, protein level for p53 and p21 was evaluated by Western blotting when NIR was silenced with two NIR-specific siRNAs in U2OS cell. As shown in Figure 6A, knockdown of NIR did not change p53 protein level but caused an increase in p21 level. To confirm p21 activation induced by NIR depletion, RT-PCR was performed to evaluate mRNA level of p21 when NIR was silenced. We show that p21 mRNA increased in NIR-depleted cells (Figure 6A). Since NIR is a HAT inhibitor, we then asked if NIR affects p53 acetylation, NIR siRNA was transfected into U2OS cell and acetylation of p53 was evaluated as described previously [43], [44]. In brief, immunoprecipitation was performed with an anti-acetyl-lysine antibody and proteins from immunoprecipitates were subjected to Western blotting and probed with anti-p53 antibody. Figure 6B shows that acetylation of p53 increased when NIR was depleted, while acetylation of the upstream binding factor (UBF) which is a transcriptional factor for polymerase I was not affected demonstrating that depletion of NIR led to enhancement of p53 acetylation. Given that p53 acetylation also activate proapoptotic genes [45] and knockdown of NIR induced apoptosis [39], we evaluated expression level of PUMA and BAX when NIR was silenced. As shown in Figure 6C, both PUMA and BAX increased when NIR was knocked down. To determine whether knockdown of NIR affect cell proliferation, colony formation was performed when NIR was depleted by siRNAs. As shown in Figure 6D, both of the number and size of the cell colonies decreased when NIR was knocked down. To quantity the effect of NIR depletion on cell growth, a cell growth curve was plotted using CCK-8 assay. As shown in Figure 6E, knockdown of NIR dramatically inhibited cell growth. Collectively, these experiments demonstrated that depletion of NIR activated p53 possibly by de-repression of p53 acetylation.

## Discussion

U3 snoRNA has been demonstrated to participate in the cleavage at A0, A1 and A2 (Figure 2A) to facilitate 18S rRNA processing [7], [8]. U3 associated protein (UTP) has been identified if the proteins meet three conditions: is nucleolar, is associated with U3 snoRNA and is required for 18S rRNA processing. In the present study, we showed that NIR can be identified as a UTP according to the above criteria. However, in addition to inhibiting 18S rRNA, depletion of NIR also caused inhibition of 28S and 5.8S rRNA processing. Thus we identify NIR as an UTP-like nucleolar protein. For the 28S rRNA biogenesis, U8 snoRNA binds 32S pre-rRNA through base-pairing with 5.8S/ITS-2 junction and 28S rRNA sequence [9], [10]. This helix formation may facilitate the correct folding of 32S rRNA which may allow the subsequent rRNA processing at sites 3, 4, 5 and T1. When processing occurs, U8 snoRNA is displaced by an intermolecular interaction between 28S and 5.8S. Disruption of U8 snoRNA inhibits 28S and 5.8S rRNA processing by inhibiting cleavage at sites 3, 4 and 5. Our data demonstrated that NIR interacted with both of 32S rRNA and U8 snoRNA, suggesting that NIR may participate in maintaining the binding between 32S rRNA and U8 snoRNA thus contributing to the correct processing from 32S rRNA to 28S rRNA. Collectively, our data suggested that NIR was involved in the cleavage at site A0, A1, A2, site 3, 4 and 5.

In the nucleolus of eukaryote, rRNA processing occurs in the form of pre-rRNP particles. The 90S pre-rRNP contains nascent 47S rRNA precursor and 40S subunit processing factors but lacks 60S subunit processing factors [7], [8]. When cleavage at site A2 is fulfilled, it is the time point for the separation of the pre-40S and pre-60S ribosome subunits. The 60S processing factors are recruited to the 32S pre-rRNA and this step initiates formation of 60S pre-ribosome subunit. Then, the two pathways proceed independently leading to the maturation of 40S and 60S subunits. In the present study, nuclear extracts were isolated from U2OS cells and subjected to sucrose gradient fractionation to fractionize the pre-40S and pre-60S rRNP particles. The result revealed that NIR sediment in both of the pre-60S and pre-40S rRNP particles. Northern blotting results showed that NIR coexisted with the 32S and 12S rRNA precursors in pre-60S rRNP particles. These results further demonstrated that NIR functions in the entire process of rRNA processing from the very early stage as formation of 41S rRNA till the maturation of 18S, 28S and 5.8S rRNAs.

Our data demonstrated that NIR is required for 28S and 5.8S rRNA processing and was associated with 32S rRNA and U8 snoRNA. Bop1, Pes1 and WDR12 are interdependent and form a PeBoW complex to play key roles in 60S ribosome subunit biogenesis and cell proliferation [21]. However, none of them has been found to bind 32S rRNA or U8 snoRNA. Whether NIR is involved in the PeBoW complex will be investigated in the future study.

Nucleolar functional disruptions have been shown to generate nucleolar stress signaling to p53. Defects in ribosome biogenesis often cause p53 accumulation and activation. However, our results and previous studies [39], [40] have shown that knockdown of NIR did not change protein levels of p53 but activated p21 transcription. Hublitz et. al [39] demonstrated that NIR interacted with p53 and repressed p53 activity. Jain and colleagues found that NIR recruits Aurora B to p53 and phosphorylates p53 at multiple sites and thus suppresses p53 activation. Given that NIR has been found to inhibit histone acetylation and inhibit transcriptional activation of p53, we proposed that NIR may also inhibit p53 acetylation. In the present study, we showed that depletion of NIR led to enhancement of p53 acetylation and activation which is evidenced by activation of p21, PUMA and BAX. We thus provide a novel mechanism for nucleolar stress induced p53 activation in which functional disruption of a ribosome biogenesis factor activates p53 through enhancing p53 acetylation without altering p53 protein level. How NIR affects p53 acetylation needs further investigation.

## Materials and Methods

### Cell culture and NIR RNAi

HeLa, H1299, U2OS, HEK293, MCF10A, MCF-7, T47D, MDA-MB231, HepG2, A549 and K562 cells were obtained from the American Type Culture Collection (ATCC) and were grown according to the instructions provided by the ATCC. Cells were incubated in a humidified atmosphere with 5% CO_2_ at 37°C. For silencing NIR expression, two siRNAs targeting NIR (NIR siRNA-1: 5′-GACCUGAACUUCCCUGAGA-3′; NIR siRNA-2: 5′-GGAUGAGGACAGGAAGCAA-3′) together with a control siRNA [39] (5′-CGU ACGCGGAAUACUUCGA-3′) were chemically synthesized (Shanghai GenePharma Co., Ltd). The synthesized siRNA was transfected into cells at a concentration of 100 nM with Lipofectamine 2000™ (Invitrogen) according to the manufacturer's instructions.

### Plasmids and antibodies

pGEM-T-U3, p-GEM-T-U8 and pCMX-Flag-NIR are described previously [36], [39]. To generate pGEM-T-U1 plasmid, cDNA fragment coding U1 snRNA was amplified by RT-PCR with the following primers: forward primer: 5′-AGCTGAATTC (EcoR I)ATACTTACCTGGCAGGG-3′, reverse primer: 5′-AGCTAAGCTT (Hind III)GGAAA GCGCGAACGCAG-3′. The PCR product was cloned into p-GEM-T-easy vector (Promega). The generated plasmid was verified by DNA sequencing. Anti NIR polyclonal antibody was described previously [39]. Anti-UBF, anti-topoisomerase I, anti-GFP, anti-p53, anti-RhoA and anti-β-actin antibodies were obtained from Santa Cruz. Anti-Flag antibody was from Sigma. Anti-acetyl-lysine antibody was from Upstate.

### Preparation of cellular fractions

Cellular fractions were obtained as previously described [46]. In brief, pelleted U2OS and HeLa cells were resuspended in ice-cold mild detergent buffer and centrifuged. The supernatant was retained as the cytoplasmic fraction. The nuclear pellets were then resuspended in 0.25 M sucrose/10 mM MgCl_2_, layered over a cushion of 0.35 M sucrose/0.5 mM MgCl_2_ and centrifuged. The resulting nuclear pellet was resuspended in 0.35 M sucrose/0.5 mM MgCl_2_ and sonicated to disrupt nuclei and release nucleoli. The sonicate was layered over a cushion of 0.88 M sucrose/0.5 mM MgCl_2_ and centrifuged to pellet nucleoli and the supernatant was retained as the nucleoplasmic fraction. Nucleoli were washed by resuspending in 0.5 ml of 0.35 M sucrose/0.5 mM MgCl_2_ and centrifugation. The nucleolar pellet was resuspended in high salt RIPA buffer containing DNase, and then sonicated and centrifuged after incubation, the supernatant was retained as the nucleolar extract and the NaCl concentration adjusted to 150 mM by adding “no salt” RIPA buffer.

### Preparation of cellular extracts and immunoprecipitation

Immunoprecipitation was performed as described previously [47]. Briefly, U2OS cell lysates were prepared in NET buffer (150 mM NaCl, 5 mM EDTA, 50 mM Tris-Cl [pH 7.5]) supplemented with 1% NP-40 and protein cocktail inhibitor. Cell lysates were incubated with anti-NIR anbibody or rabbit IgG crosslinked protein A sepharose beads (Amersham Biosciences) for 4 h at 4°C. After washing twice with NET+1% NP-40 and twice with NET buffer, the precipitated proteins were subjected to Western blotting and co-precipitated RNA was isolated with Trizol reagent (Invitrogen).

### Immunoblotting

Proteins were separated on SDS-PAGE and transferred onto PVDF membranes (Amersham Biosciences). Blots were probed sequentially with corresponding antibodies and HRP-conjugated secondary antibodies. Immunocomplexes were detected with ECL Western blot Detection Reagent (GE Healthcare) before exposure to X-ray film.

### Northern blotting and RT-PCR

For detection of 32S rRNA, Northern blotting was performed as described previously [48]. In brief, RNA was extracted with Trizol reagent (Invitrogen) and loaded onto 1% agarose-glyoxal (NorthernMax®-Gly kit, Ambion) and blotted onto BrightStar®-PLUS positively charged Nylon membrane (Ambion). The previously described ITS-2 DNA oligonucleotide (human specific) [21]: 5′-GCGGCGGCAAGAGGAGGGCGGACGCCGCCGGGTCTGCGCTTAGGGGGA-3′ was biotin-labeled with Biotin 3′ End DNA Labeling Kit (PIERCE) and used to probe 32S rRNA precursor. Hybridization was performed as described previously [48]. The hybridized 32S rRNA was detected with BrightStar® BioDetect™ Nonisotopic Detection Kit (Ambion).

To visualize U3 snoRNA, U8 snoRNA or U1 snRNA, Northern blotting was performed as described previously [36] with minor modifications. In brief, RNA sample was loaded onto 7% polyacrylamide/8.3 M urea gel and blotted onto BrightStar®-PLUS positively charged Nylon membrane (Ambion) by electroblotting (Bio-Rad). The U3 or U8 snoRNA or U1 snRNA-specific RNA probe was labeled with biotin-UTP using Riboprobe® System (Promega) with Sal I-linearzed pGEM-T-U3, pGEM-T-U8 or pGEM-T-U1. Hybridization was carried out for 16 h at 65°C after 3 hr's prehybridization. After extensive washing, the detection was performed with BrightStar® BioDetect™ Kit (Ambion) according to the instruction.

p21 mRNA level was determined by RT-PCR as previously described [40]. The primers used to amplify p21 and GAPDH were as previously described [49].

### Analysis of newly synthesized rRNA with metabolic labeling

For 18S and 28S rRNA processing analysis, pulse-chase labeling was performed as described previously [17]. In brief, 72 hours after transfection of chemically synthesized siRNA, U2OS cells were pre-incubated in methionine-free medium for 15 min and then incubated in medium containing 50 µCi/ml L-[*methyl*-^3^H] methionine (PerkinElmer Life Sciences) for 30 min. Cells were then changed to medium containing 15 µg/ml of non-radioactive methionine. Total RNA was isolated with Trizol reagent (Invitrogen) at different time points. The [*methyl*-^3^H] methionine labeled RNAs were resolved on a 1% agarose–formaldehyde gel and detected by fluorography. For 5.8S rRNA labeling, pulse-chase experiment was performed as described previously [2]. In brief, U2OS cells were transfected with siRNAs. At 72 hrs post-transfection, cells were incubated with 3 µCi/ml [5, 6-^3^H] uridine (PerkinElmer Life Sciences) for 30 min. After two brief rinses, cells were changed to nonradioactive medium and total RNA was isolated with Trizol reagent at different time points. Equal amount of RNA was resolved on a 10% polyacrylamide/7.5 M urea gel and newly synthesized 5.8S rRNA was detected by fluorography.

### Sucrose density gradient fractionation

Nuclear extracts were fractionated as described previously [16] with minor modifications. In brief, cells were swollen in ice-cold hypotonic lysis buffer (10 mM Tris [pH 7.4], 10 mM KCl, 2 mM MgCl, 0.05% Triton X-100, 1 mM EGTA, 1 mM DTT, 40 mg/ml of phenylmethylsulfonyl fluoride, 10 mg/ml of protease inhibitor cocktail [Sigma]). The nuclei pellet was collected by centrifugationm at 700 *g* for 5 min. The nuclear lysate were extracted in 25 mM Tris (pH 7.5), 100 mM KCl, 1 mM DTT, 2 mM EDTA, 0.1% NP-40, 1 mM NaF, 40 mg/ml of phenylmethylsulfonyl fluoride, 10 mg/ml of protease inhibitor cocktail, 0.1 U/ml of RNasin (Promega) and sonicated. The nuclear lysate was overlaid on 10 to 40% (wt/wt) sucrose gradients in 25 mM Tris (pH 7.5), 100 mM KCl, 1 mM DTT, 2 mM EDTA and centrifuged at 36,000 rpm for 3 h at 5°C in a Beckman SW41Ti rotor. The gradients were collected downward and the absorbance at 254 nm was measured [16].

### Immunofluorescence

Cells were plated on coverslips in 6-well plates one day before harvest. Double indirect immunofluorescence was performed with the monoclonal anti-1A6/DRIM antibody 6D9 and a polyclonal antibody directed against NIR after cells were fixed. 1A6/DRIM specific immunocomplexes were detected with TRITC-conjugated goat anti-mouse IgG and NIR specific immunocomplexes were detected with FITC-conjugated goat anti-rabbit IgG. Immunofluorescence signals were recorded by confocal laser scanning microscopy (Leica TCS-ST2).
